# Proteomic characterization of neuromelanin granules isolated from human *substantia nigra* by laser-microdissection

**DOI:** 10.1038/srep37139

**Published:** 2016-11-14

**Authors:** Sarah Plum, Simone Steinbach, Johannes Attems, Sharon Keers, Peter Riederer, Manfred Gerlach, Caroline May, Katrin Marcus

**Affiliations:** 1 Medizinisches Proteom-Center, Ruhr-University Bochum, Bochum, Germany; 2 Institute of Neuroscience and Newcastle University Institute for Ageing, Newcastle University, Newcastle upon Tyne, United Kingdom; 3 University Hospital Wuerzburg, Center of Mental Health; Clinic and Policlinic for Psychiatry, Psychosomatics and Psychotherapy, Fuechsleinstrasse 15, D-97080 Wuerzburg, Germany; 4 Center of Mental Health, Department of Child and Adolescent Psychiatry, Psychosomatics and Psychotherapy, University Hospital of Würzburg, University of Würzburg, Würzburg, Germany

## Abstract

Neuromelanin is a complex polymer pigment found primarily in the dopaminergic neurons of human *substantia nigra*. Neuromelanin pigment is stored in granules including a protein matrix and lipid droplets. Neuromelanin granules are yet only partially characterised regarding their structure and function. To clarify the exact function of neuromelanin granules in humans, their enrichment and in-depth characterization from human *substantia nigra* is necessary. Previously published global proteome studies of neuromelanin granules in human *substantia nigra* required high tissue amounts. Due to the limited availability of human brain tissue we established a new method based on laser microdissection combined with mass spectrometry for the isolation and analysis of neuromelanin granules. With this method it is possible for the first time to isolate a sufficient amount of neuromelanin granules for global proteomics analysis from ten 10 μm tissue sections. In total 1,000 proteins were identified associated with neuromelanin granules. More than 68% of those proteins were also identified in previously performed studies. Our results confirm and further extend previously described findings, supporting the connection of neuromelanin granules to iron homeostasis and lysosomes or endosomes. Hence, this method is suitable for the donor specific enrichment and proteomic analysis of neuromelanin granules.

Neuromelanin (NM) is a black-brownish pigment that mainly accumulates in dopaminergic neurons in the *substantia nigra pars compacta* (SNpc). In these dopaminergic neurons NM is associated with a protein matrix as well as lipid droplets and stored in intracellular granules^1^. So far NM granules cannot be identified in human newborns, but seem to start forming in childhood presumably from the age of three years. From then on an increase in amount and density of NM granules can be observed during normal aging^2^,^3^. The physiological function of NM granules is controversial discussed in literature. Besides an involvement of NM granules in cell iron homeostasis, a neuroprotective function by sequestration of toxic substances e.g. of environmental chemicals and metals or byproducts of dopamine synthesis is postulated^4^,^5^. Diametrically oppose neurotoxic effects of NM through production of oxygen and nitrogen species^6^,^7^ as well as microglia or dendritic cell activation^8^,^9^,^10^ have been discussed due to the fact that especially NM granules containing neurons are prone to the selective degeneration in Parkinson’s disease^5^,^11^. A specificity of NM granules is, that they do not form in common laboratory animals like mice^12^. Therefore, the described hypotheses about the function of NM granules in humans are based on experiments in cell culture and animals exposed to synthetic NM pigment or isolated human NM pigment^6^,^7^,^8^,^9^,^10^.

In order to understand the function of NM granules in human individuals, in-depth characterization of the NM granule content derived directly from human brain tissue is needed. Previous analyses of SNpc tissue in the context of Parkinson’s disease have predominately involved whole tissue lysates^13^ because the enrichment of sub-fractions or even subcellular fractions, such as NM granules, often requires larger quantities of human brain tissue than the amounts available. However, analysis of subcellular entities permits the identification of proteins that are less abundant and enables a more focused view of NM granules-related processes. Our group has previously developed methods based on density-gradient centrifugation with reduced tissue requirements to enrich NM granules, as well as synaptosomes for proteomic analysis^14^. Mass spectrometric analysis of those enriched NM granules led to the identification of NM granules specific proteins for the first time. Based on our proteomic data we hypothesized the generation of NM granules to resemble lysosome formation^1^. A limiting factor of those studies was obtaining sufficient numbers of individual high quality SNpc samples in required amounts (0.15 g, at least a half SNpc) to perform one experiment^14^. Due to this, only studies with small sample sizes could be performed which reduces statistical power of the experimental approach.

In order to overcome these limitations, laser microdissection (LMD) was optimized using thin sections of human *substantia nigra* (SN) tissue. Those are common in pathological studies and thus more readily available. In this paper, we present the successful application of LMD to isolate NM granules and their subsequent characterization using proteomic analysis. This enables a substantial reduction of the required tissue amounts and allows further insights regarding NM granules development and function.

## Materials and Methods

### Ethics statement

The use of human post mortem brain tissue was approved by the ethics committees of the Ruhr-University Bochum, Germany (file number 4760-13) and the University of Würzburg, Germany (file number 78/99) accordance with German guidelines and regulations. In case of tissue from the Netherlands Brain Bank, all material has been collected from donors for or from whom a written informed consent for a brain autopsy and the use of the material and clinical information for research purposes had been obtained by the Netherlands Brain Bank. Tissue from the Newcastle Brain Tissue Resource was obtained in accordance with the approval of the joint Ethics Committee of Newcastle, United Kingdom, and North Tyneside Health Authority, United Kingdom, with written informed consent and following Newcastle Brain Tissue Resource brain banking procedures.

### Subjects

Cryopreserved *postmortem* SN sections from five human subjects with no history of neurological or neurodegenerative diseases and lacking neuropathological abnormalities were provided by the Brain Bank Center Würzburg, the Newcastle Brain Tissue Resource, UK and the Netherlands Brain Bank, Netherlands Institute for Neuroscience, Amsterdam. Neuropathological diagnoses were assigned using accepted international neuropathological criteria including neuritic Braak stages, Thal amyloid phases, CERAD scores, NIA-AA scores and McKeith criteria.

### Cryosectioning for laser microdissection

The frozen midbrains were stored at −80 °C and sectioned at the level of the SN, which can be clearly identified by its dark color^15^. With a Microm HM550 cryostat (Thermo Scientific, Dreieich, Germany) combined with a fixed knife holder (Leica Biosystems, Nußloch GmbH, Nußloch, Germany), slices were cut at −18 °C object temperature and −20 °C chamber temperature. The 5, 10 or 20 μm thick sections were placed on 1.0 PEN membrane glass slides that were optimized for LMD (Carl Zeiss Microscopy GmbH, Göttingen, Germany).

### Sampling using laser microdissection

Using the bright field microscope of LMD (PALM Micro Beam, P.A.L.M.-System, Carl Zeiss Microscopy GmbH) NM granules can be observed within the SNpc without staining because of their black-brownish color. First, under bright field microscopy, the tissue slices were screened for NM granules at 400x magnification, and these granules were marked using software supplied by the manufacturer (PALMRobo 4.6 pro, Carl Zeiss Microscopy GmbH). Once all NM granules in a tissue section were marked, a non-adhesive microtube cap (MicroTube 500, Carl Zeiss Microscopy GmbH) filled with 50 μL of distilled water was applied to the instrument and placed over the tissue slice. First, NM granules were cut and catapulted with a laser into the sample cap. Afterwards, the tube was carefully closed and stored at −80 °C until further usage. Tissue depleted of NM granules was collected as control in a fresh tube as contamination control and treated like the NM granules samples (further referred to as “control”).

### Proteomic analysis using mass spectrometry

Prior to tryptic digestion, the NM granules and control samples were adjusted with 2% (w/v) RapiGest™ SF Surfactant (Waters GmbH, Milford, MA, USA) to a final concentration of 0.1% RapiGest™. After incubation in an ultrasonic bath for 1 min, the samples were briefly centrifuged. The digestion protocol was performed as previously described^15^. Briefly, 250 mM dithiothreitol was added and the samples were incubated for 30 min at 60 °C. Then, 0.55 M iodoacetamide was added and the samples were incubated for another 30 min in the dark. Trypsin was diluted in 50 mM ammonium bicarbonate and was added 1:25 (v/v) to the samples. Subsequently, digestion was performed for 4 h at 37 °C. The reaction was stopped via the addition of 10% trifluoroacetic acid (TFA) and incubation for 30 min at 37 °C. After 15 min centrifugation at 20,800 × g_(av)_, the supernatant was transferred to a new sample vial and completely dried in a SpeedDry vacuum concentrator (RVC 2-25 CDplus, Martin Christ Gefriertrocknungsanlagen GmbH, Osterode, Germany). The peptides were stored in 20 μL of 0.1% TFA and maintained at −80 °C until the mass spectrometric measurements could be performed. The peptide concentrations were determined via amino acid analysis as previously described^14^. Liquid chromatography tandem mass spectrometry (LC-MS/MS) analyses were performed with 200 ng of the sample per run using an UltiMate 3000 RSLC nano LC system (Dionex, Idstein, Germany) coupled to an Orbitrap Elite mass spectrometer (Thermo Fisher Scientific, Bremen, Germany). Moreover, equal amounts of each sample were taken to prepare a mixture, which was measured in between the samples allowing analyzation of technical variances during measurements.

The peptides were first loaded on a capillary pre-column (Dionex, 100 μm × 2 cm, particle size 5 μm, pore size 100 Å) and washed for 7 min with 0.1% TFA. The pre-column was subsequently connected with an analytical C18 column (Dionex, 75 μm × 50 cm, particle size 2 μm, pore size 100 Å). Peptide separation was performed with 400 nL/min flow rate with a gradient that was initiated with 95% A (0.1% formic acid) and 5% B (84% acetonitrile, 0.1% formic acid) with increasing amounts of B (up to 40% within 95 min). The concentration of B was then increased to 95% within 2 min and maintained for 3 min. Afterwards, the column was again adjusted to 5% B. The LC system was directly coupled with a nano electrospray ionization source (Thermo Fisher Scientific) to the Orbitrap Elite mass spectrometer. The system operated with a scan range from 300 to 2,000 *m/z* with a resolution of 30,000 and 500 ms maximum acquisition time. From each full scan, the 20 most intensive ions were selected for low-energy collision induced dissociation (CID) with 35% collision energy and 50 ms maximal acquisition time. After fragment ion (MS/MS) scans, the mass to charge (*m/z*) values of the precursor masses were maintained for 35 s on a dynamic exclusion list. The mass spectrometry raw data have been deposited to the ProteomeXchange Consortium via the PRIDE^16^ partner repository with the dataset identifier PXD004987 and 10.6019/PXD004987.

### Data analysis

Raw files derived from the measurements were analyzed using Proteome Discoverer 1.4 (Thermo Fisher Scientific, Bremen, Germany). For these analyses, a workflow template was designed using the Mascot search algorithm (Matrix Science, London, UK, version 2.2.0) for protein identification. The peptide mass tolerance was set to 5 ppm, and the fragment mass tolerance was 0.4 Da. The enzyme settings were set to “trypsin” and one possible missed cleavage site. Methionine oxidation was set as a variable modification and carbamidomethylation at cysteine was set as fixed modification. All data were searched against the UniProt/SwissProt decoy database (release 2015_05 of 29.04.2015; 548,454 entries without decoys) with a restriction to “*Homo sapiens*”. Resulting files were deposited along with the raw files to the ProteomeXchange Consortium via the PRIDE^16^ partner repository with the dataset identifier PXD004987 and 10.6019/PXD004987.

The spectral index calculations were subsequently performed as described^17^,^18^. To reduce false-positive identifications the false discovery rate (FDR) was set on peptide spectrum match (PSM) level to <0.01, the PSM were exported from PIA (https://github.com/mpc-bioinformatics/pia) and further processed using the “pivot table” function in Microsoft Excel, which yielded a table of the spectral counts for every peptide that belonged to a certain protein in a sample. Here, only peptides assigned to be unique by PIA were used for protein identification and further relative quantification. Using this strategy, no decoy entries were left, hence protein level FDR was 0. The resulting protein table was then filtered, and only proteins identified in at least four of five NM granules or in three of four control samples with a t-test p-value <0.05 and a significant fold change were accepted for subsequent analysis. For this, technical variance was estimated by analyzing mixture measurements with spectral counting as described above. Proteins identified in at least two of six measurements were used to calculate technical variances by dividing the mean of the spectral indices through the standard deviation. The mean of the protein specific variation coefficients was 0.2644 and was multiplied with 4 to estimate which fold change would be accounted as differential by biological means and not differential due to technical variance. The resulting fold change cut off value was 1.058.

Subsequently, the list of proteins overrepresented in NM granules compared to the control (t-test<0.05; fold change <1.058) was analyzed using Ingenuity^®^ pathway analysis (http://www.ingenuity.com, Qiagen, Redwood City, CA 94063, USA) to roughly determine their function and localization. Ingenuity^®^ pathway analysis is a web-based software application for the analysis, integration, and interpretation of data. Proteins were uploaded to assign localization and function basing on a knowledge base implemented in the software.

Identified proteins were also compared with proteins identified from NM granules enriched by density gradients^1^,^14^,^19^. Moreover, the proteins were further characterized regarding their function and localization via the analysis of Gene Ontology data deposited at www.uniprot.org and via functional annotation clustering using DAVID^20^,^21^.

Additionally, resulting p-values were adjusted using R according to the method of Benjamini and Hochberg to control the false discovery rate of the t-test and identify highly significant differential proteins^22^,^23^.

The resulting data are shown in Supplement Table 1.

## Results

### Established laser microdissection settings for neuromelanin granules isolation

**Figure 1:**
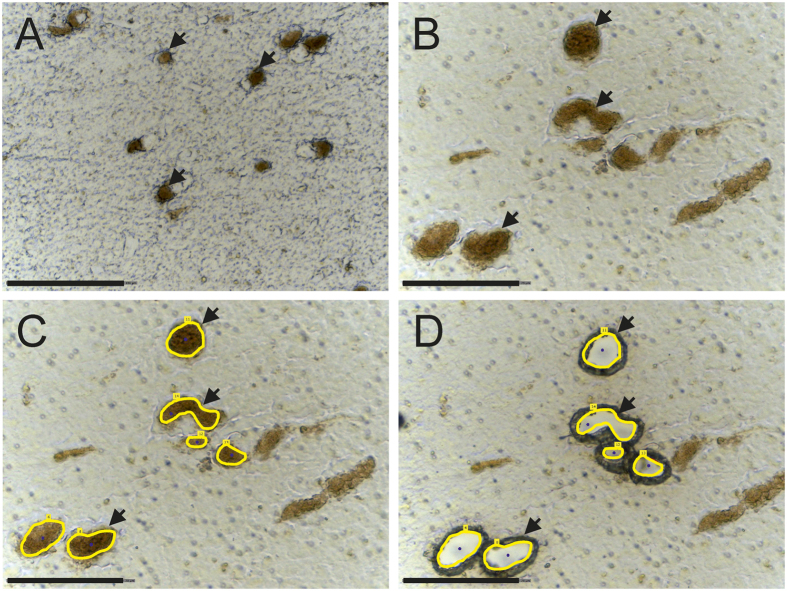
Substantia nigra tissue mounted on a PEN membrane slide at (**A**) 200x magnification and (**B**–**D**) 400x magnification. Neuromelanin granules (examples indicated by arrows) can be identified as black-brownish structures. (**C**) shows the tissues with marked neuromelanin granules (yellow line) for the LMD and (**D**) after cutting and catapulting neuromelanin granules in a tube cap. Scale bars in A = 150 μm and B–D = 75 μm.

First, the parameters for NM granules isolation were tested using *postmortem* tissues from human donors without neurological or neurodegenerative diseases and lacking neuropathological abnormalities. In order to evaluate the optimal parameters for laser microdissection, 5, 10 and 20 μm thick sections were mounted on PEN membrane slides. Further staining was omitted because the NM granules are naturally black-brownish colored and standard histological staining methods might have an effect on subsequent proteomic analysis^24^,^25^. Figure 1 shows SN tissue at 200x (A) and 400x (B–D) magnification. Using 200x magnification (A), it was possible to identify NM granules, cut 5, 10 and 20 μm thick sections and catapult NM granules into the micro tube lid. The analysis at 400x magnification of the fractions cut at 200x magnification indicated that regions marked for isolation may have also included neuronal tissue other than NM granules. Thus, 400x magnification was chosen for cutting to reduce contamination by surrounding tissue, but with these settings it was not possible to cut 20 μm thick sections. In the following experiments, NM granules were enriched from 10 μm thick SN slices using 400x magnification.

### Identified proteins from neuromelanin granules derived by laser microdissection

To ensure that sufficient material was available for LC-MS/MS analysis, at least 550,000 μm^2^ of NM granules were collected from an average of six 10 μm SN sections of each tested tissue. After tryptic digestion of NM granule proteins, the peptide concentration was estimated to be between 0.04 and 0.07 μg/μl. LC-MS/MS measurements and data analysis were performed resulting in the identification of 214 significantly differential proteins (t-test p-value <0.05) in NM granules compared to control. Of these, 48 were underrepresented and 166 proteins were overrepresented in NM granules.

To further analyze the quality of our LMD based enrichment strategy, the identified proteins were compared with published data^1^,^19^. 50 of the 73 proteins identified by Tribl *et al*. in NM granules enriched using sucrose and Percoll^®^ density gradients^1^ were identified in at least four of five LMD processed NM granules samples indicating an overlap of 68%. 17 of these proteins (23%) were significantly overrepresented in NM granules in comparison to control. We additionally compared proteins identified in the study published by Plum and co-workers in 2013 based on density gradient centrifugation^14^. Here, we identified 277 proteins related to NM granules, of which 203 (73%) proteins were also identified in at least four of five LMD-enriched NM granules, 32 of these proteins were significantly overrepresented in NM granules compared to control. As most of the previously identified NM granules related proteins could be identified with our approach, it is reasonable to conclude that laser microdissection is an optimally suited enrichment method for NM granule.

### Functional and biological character of proteins identified in neuromelanin granules

**Figure 2:**
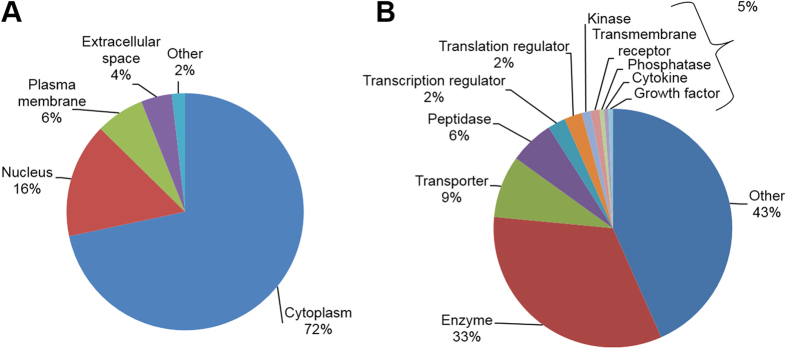
Diagrams showing the distribution of proteins overrepresented in neuromelanin granules compared to control according to their localization (**A**) or function (**B**). A large number of neuromelanin granule-associated proteins are assigned to the cytoplasm. Furthermore, 33% of the proteins have enzymatic functions and 8.4% are transporters.

The cellular distributions and functions of the 166 significantly overrepresented proteins in NM granule samples using Ingenuity^®^ pathway (http://www.ingenuity.com) revealed the following: 70% of these proteins are cytoplasmic proteins, followed by proteins associated with nuclei (15.7%; Fig. 2A). In terms of function 33% of the proteins are known to have enzymatic functions and 8.4% of the proteins are transporters (Fig. 2B).

In-depth analysis of our data indicated iron binding proteins in NM granules, such as sideroflexin-1 or serotransferrin, being significantly (p-value <0.05) overrepresented in NM granules. Ferritin light chain previously described to be a component of NM granules^19^ as well as heavy chains were identified but with a t-test p-value >0.05. Interestingly, both alpha- and beta-synuclein were identified unequivocally significantly overrepresented in NM granules. Additionally, proteins related to lysosomes or endosomes such as lysosomal alpha-glucosidase, sialate O-acetylesterase, mammalian ependymin-related protein 1, dipeptidyl peptidase II, Cathepsin (B, D and Z), syntaxin 8 or serotransferrin showed higher abundance in NM granules.

19 proteins significantly overrepresented in NM granules are associated with lysosomes (DAVID p-value 5.89 e^−11^, cluster 4 enrichment score 4.35), hence supporting the hypothetical link between lysosomes and NM granules as previously hypothesized^1^.

Besides, 20 proteins were related to the Golgi complex (DAVID p-value 0.0075, cluster 9 enrichment score 1.66) and 25 proteins were found to be associated with the endoplasmic reticulum (DAVID p-value 3.99 e^−4^, cluster 5 enrichment score 2.67).

The origin of NM pigment is not clear so far. The pigment is believed to originate from the tyrosinase-catalyzed oxidation of dopamine similar to the melanin synthesis in the skin^26^,^27^,^28^,^29^. Therefore, we specifically searched our data for enzymes being involved in this pathway. In accordance with immunohistochemical and Western blot analysis of the human SN^30^,^31^ tyrosinase could not be identified in our study; neither in the NM granules nor in the control. Instead, tyrosine hydroxylase which catalyzes the reaction from L-tyrosine to L-3,4-dihydroxyphenylalanine (L-DOPA) was identified unambiguously: it seems to be more than twofold overrepresented in NM granules, but the results were not significant (t-test p = 0.226) in our preliminary study with only 5 individual samples. Dopa decarboxylase, which *inter alia* catalyzes the decarboxylation of L-DOPA to dopamine, was detected significantly (t-test p-value = 0.029) and more than eight times overrepresented in NM granules. Furthermore, a protein important for dopamine degradation, amine oxidase A (MAO-A) was identified in NM granules but there was no significant difference besides it seemed slightly underrepresented compared to control (fold change -1.3, t-test p-value 0.56).

## Discussion

Here, we present a new strategy to isolate NM granules from cryopreserved *postmortem* SN sections from human subjects with no history of neurological or neurodegenerative diseases and lacking neuropathological abnormalities. The approach was successfully applied by using LMD and subsequent LC-MS/MS analysis for proteome analysis and will in future permit further in-depth characterization of NM granules from both neuropathological healthy donors as well as patients suffering from neurodegenerative diseases. Furthermore, LMD allows the enrichment of other cellular or subcellular structures such as neurons^15^, Lewy bodies^32^,^33^ or amyloid beta plaques^34^. Thus, it can be thought of combining different protocols to co-enrich e.g. NM granules and Lewy bodies from the same tissue.

Compared to previous enrichment protocols, which were applicable to at least 0.15 g tissue^14^, the LMD based enrichment strategy presented in this paper allows to use much lower amounts of tissue. As thin tissue sections were used, total amounts of tissue can only be estimated. In average tissue slices were 2 × 1 cm in size and tissue was sliced to 10 μm thickness. This results in a volume of 0.002 cm^3^. Brain tissue density has been described to be about 1.04 g/cm^3^ 35. A maximum of 8 thin sections were used to obtain sufficient amounts of neuromelanin granules, hence roughly up to 16.6 mg tissue was used compared to at least 150 mg up to 1 g brain tissue used a priori^1^,^14^.

We used LC-MS/MS to identify proteins associated with NM granules to easily compare the enrichment data with our previously published data^1^,^14^,^19^. However, the presented method could also be combined with other analytical strategies, such as anti-melanin ELISA^36^, e.g., combined with a second ELISA to analyze whether specific proteins are attached to the NM granules.

The overlap of identified proteins compared to previously published data was between 68% and 73%. Remaining differences could be due to technical variances, resulting from different enrichment methods (density gradient based versus LMD) as well as alterations in the measurement systems. E.g. Tribl *et al*. used an ion trap mass spectrometer several years ago with less resolution, accuracy and sensitivity compared to the orbitrap system used in the presented work^1^. However, it is more likely, that the reported differences reflect biological variances, as tissues from different human donors were used in the different studies varying e.g. in country of origin, age or gender. This hypothesis needs to be verified in future studies with higher case numbers. Anyhow, the protein overlap between this study and former studies of Tribl *et al*. as well as Plum *et al*. was satisfactory, clearly demonstrating the applicability of this new established LMD-based enrichment method. We identified many of the previously reported NM granules associated proteins e.g. both ferritin light and heavy chain as well as sideroflexin-1 indicating a connection of NM granules to iron ion homeostasis.

**Figure 3:**
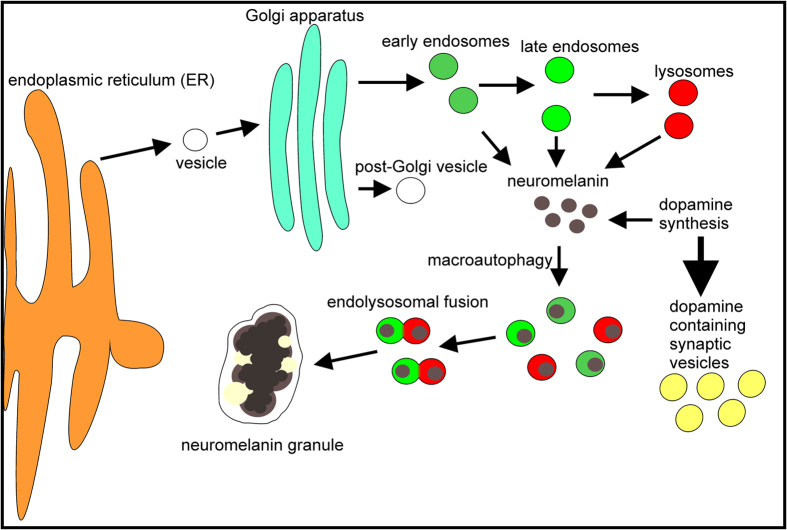
Scheme of putative neuromelanin granules genesis. Proteins identified in neuromelanin granules were analyzed by Gene Ontology term enrichment and mapped according to their occurrence in subcellular compartments. The distribution of protein locations supports the theory that neuromelanin forms in the cytoplasm at physiological temperature and pH as a side product of dopamine synthesis and is incorporated by endosomes or lysosomes. Because neuromelanin is non-degradable, neuromelanin granules could form during aging by the fusion of many neuromelanin-laden endosomes and lysosomes in multivesicular bodies.

In addition to NM pigment, NM granules consist of approximately 15% amino acids and approximately 20% lipids^4^. Assuming, that the protein content is a result of protein sequestration during NM synthesis, the characterization of proteins contained in NM granules provides insights regarding NM development. The analysis revealed 166 proteins significantly overrepresented in NM granules compared to control. By utilization of the Gene Ontology annotations 19 proteins of the differentially expressed proteins could be related to lysosomes, which underlines the lysosomal relationship of NM granule which was already previously described^37^. Previously knowledge^2^,^3^ combined with these new findings lead to the hypothetical process describing NM granules genesis depicted in Fig. 3.

After formation, NM could subsequently be macro-phagocytized and packed in endosomes or lysosomes for degradation. Since NM is non-degradable aggregation in endosomes or lysosomes is likely. Fusion of NM containing endosomes might finally result in growing NM granules surrounded by a double membrane and might explain age depending NM granules formation (1,8). Furthermore, an assumed fusion of NM granules with endolysosomes might explain identification of mitochondrial related proteins in NM granules.

## Summary and Outlook

Due to the fact that SN tissue is very valuable and challenging to obtain, the presented LMD strategy offers a major advantage compared with other NM granules enrichment strategies, because only a few thin tissue sections are required instead of an entire SN. Future studies could therefore investigate for the first time higher case numbers from healthy donors for obtaining a better statistical significance and deeper insights into the biological variance, including ethical variations. Moreover, analysis of different age groups could lead to deeper insights in NM granules genesis and function. Besides, NM granules from cases with neurodegenerative diseases e.g. Parkinson’s disease could be analyzed. Further, other cellular or subcellular structures such as neurons^15^ or Lewy bodies^32^,^33^ could be enriched from the same tissue slices allowing a thorough analysis.

Performing differential studies comparing proteins isolated from human subjects with no history of neurological or neurodegenerative diseases and lacking neuropathological abnormalities and subjects with neurodegenerative diseases could lead to a better understanding of the role of NM granules during neurodegeneration.

## Additional Information

**How to cite this article**: Plum, S. *et al*. Proteomic characterization of neuromelanin granules isolated from human *substantia nigra* by laser-microdissection. *Sci. Rep*. **6**, 37139; doi: 10.1038/srep37139 (2016).

**Publisher’s note**: Springer Nature remains neutral with regard to jurisdictional claims in published maps and institutional affiliations.

## Supplementary Material

Supplementary Information

Supplementary Table S1
