# Supplementary material for: Proteomic characterization of neuromelanin granules isolated from human substantia nigra by laser-microdissection
Source: Sci Rep. 2016 Nov 14;6:37139. doi: 10.1038/srep37139 (PMC5107900; doi:10.1038/srep37139)
Supplement: Supplementary Information [file srep37139-s1.pdf]

Supplementary information

Proteomic characterization of neuromelanin granules isolated from human *substantia nigra* by laser-microdissection

Sarah Plum[1], Simone Steinbach[1], Johannes Attems[2], Sharon Keers[2], Peter Riederer[3], Manfred Gerlach[4], Caroline May[1¶] and Katrin Marcus[1¶*]

Supplement table 1: In worksheet 1: identified in LMD samples proteins identified in neuromelanin granules and *substantia nigra* depleted of neuromelanin granules samples are shown and compared with published data. (See Excel file). identified in # NM samples: number of neuromelanin granules samples the protein was identified in (max. 5 samples); identified in # SN depleted of NM samples: number of *substantia nigra* depleted of neuromelanin granules samples the protein was identified in (max. 4 samples); moreover, averaged normalized peptide spectrum matches counts were assigned and resulting ratio of neuromelanin granules to *substantia nigra* depleted of neuromelanin granules samples shown. Additionally, a p-value basing on a student's t-test has been assigned for statistical significance of identified differences between the sample groups. Only proteins with a p-value below 5% and identified in 4/5 respectively ¾ of the samples from one group were accepted as significantly different and thus assigned as over- or underrepresented. Besides general information (Uniprot protein accession, protein name, GeneOntology information) proteins were compared with previously published studies to analyze the overlap between the studies (see rank in list from Tribl *et al.* 2005/ Plum *et al.* 2013). Moreover, results of the DAVID analysis are shown in the second worksheet.

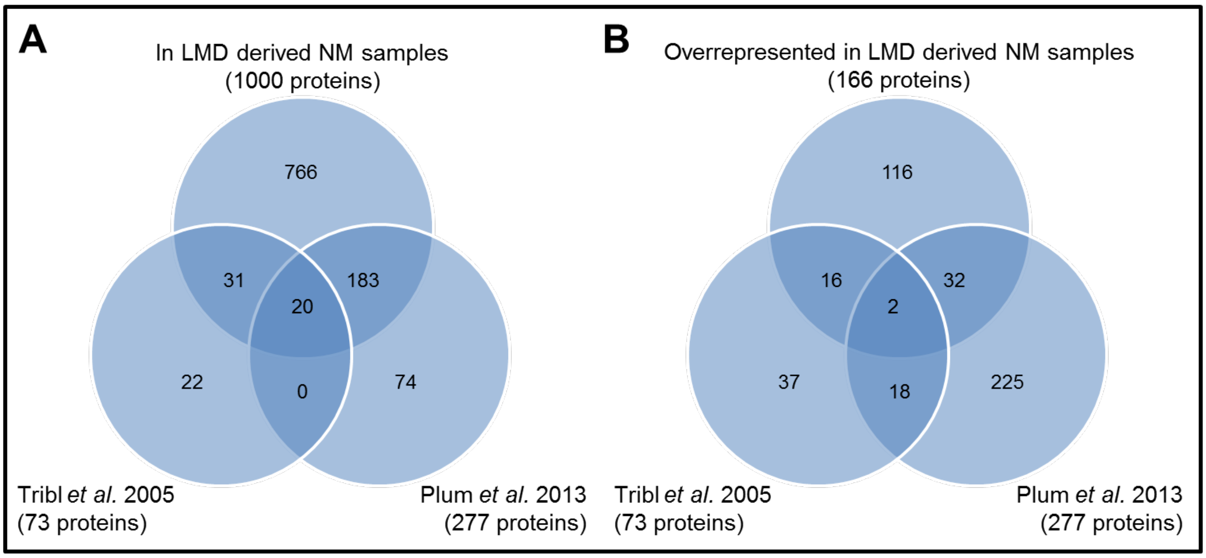

Supplement figure 1: Venn diagrams showing the protein identifications associated with neuromelanin granules. In A, all proteins identified in at least 4/5 neuromelanin granules samples were compared with previously published data (Tribl *et al.* 2005, Plum *et al.* 2013). In B, only proteins identified significantly overrepresented in neuromelanin granules samples compared to control samples were collated with previously published data.
